# Development and validation of a nomogram to predict the risk of post-stroke complex regional pain syndrome

**DOI:** 10.3389/fnagi.2025.1577256

**Published:** 2025-05-07

**Authors:** Qian Xie, Qing Song, Jianling Deng, Xuanling Cheng, Aiguo Xue, Shuxiong Luo

**Affiliations:** ^1^Department of Tuina, Dongguan Hospital of Traditional Chinese Medicine, Guangzhou University of Chinese Medicine, Dongguan, China; ^2^Department of Acupuncture and Moxibustion, Dongguan Hospital of Traditional Chinese Medicine, Guangzhou University of Chinese Medicine, Dongguan, China

**Keywords:** post-stroke complex regional pain syndrome, stroke, nomogram, prediction model, LASSO

## Abstract

**Objective:**

This study aims to assess risk factors and build a nomogram model to facilitate the early recognition of post-stroke complex regional pain syndrome (CRPS).

**Methods:**

A total of 587 stroke patients admitted to Dongguan Hospital of Guangzhou University of Traditional Chinese Medicine from September 2021 to October 2024 were initially included in this study. After exclusions, 376 patients were selected. Among these, there were 90 patients with post-stroke CRPS, while the non-stroke CRPS group consisted of 286 patients. Feature selection and optimization to generate the predictive model and nomogram were performed using LASSO regression and multivariable logistic regression analysis. We also utilized calibration plots, receiver operating characteristic (ROC) curves, decision curves (DCA), and clinical impact curves (CIC) for model validation.

**Results:**

LASSO regression analysis and multivariate logistic regression identified gender, age, NIHSS score, cervical spondylosis, sleep disorders, fasting blood glucose (FBG), and albumin (ALB) as significant predictors. The nomogram model showcased reliable predictive effectiveness, achieving an area under the curve (AUC) of 0.858 (95% CI, 0.801–0.915). Both DCA and CIC demonstrated that the nomogram model holds substantial clinical utility.

**Conclusion:**

This study has developed a novel predictive model for post-stroke CRPS, providing a valuable tool to facilitate the early detection of high-risk patients in a clinical environment.

## 1 Introduction

Complex regional pain syndrome (CRPS) is a complex pain disorder marked by autonomic dysfunction and inflammation (Bruehl, 2015). Evidence suggests that post-stroke CRPS predominantly manifests as Type I CRPS (Merskey, 1986), which is also known as shoulder-hand syndrome (SHS).

At present, research on post-stroke CRPS is sparse among scholars both domestically and internationally (Eldufani et al., 2020; Marinus et al., 2011; Taylor et al., 2021; Urits et al., 2018). The definitions, pathophysiology, treatment, and prognosis of post-stroke CRPS remain subjects of controversy, and protocols for preventing post-stroke CRPS have not yet been established. Findings suggest that the rate of post-stroke CRPS can be as high as 50.0% (Han et al., 2014; Katsura et al., 2022; Kim et al., 2020), making it one of the prevalent complications of stroke and a significant factor contributing to disability in stroke patients. It primarily manifests as restricted movement and pain in the shoulder, hand, and wrist, along with symptoms such as allodynia, swelling, changes in sweating, elevated temperature, and, in severe cases, irreversible loss of hand function (Harden et al., 2013). This condition markedly influences patients’ physical and psychological health and, subsequently, their quality of life, leading to increased disability and higher public healthcare costs. Research has reported that early treatment can lower the likelihood of developing CRPS (Kondo et al., 2001). Therefore, timely recognition of high-risk populations for post-stroke CRPS, combined with proactive and effective preventive interventions, could lead to reduced hospital stays and treatment costs while improving patients’ quality of life (Elsamadicy et al., 2018).

Prior meta-analyses have shown that factors linked to the onset of post-stroke CRPS include being female, left-sided hemiparesis, shoulder subluxation, spasticity, the upper limb’s distal Brunnstrom stage, and scoring low on the Barthel index (Su et al., 2021). However, there are currently no studies that comprehensively assess various risk factors for post-stroke CRPS using a nomogram model, which fails to meet clinical needs. Therefore, developing a risk prediction model for post-stroke CRPS specifically tailored to stroke patients holds significant clinical importance. A nomogram is a graphical tool composed of line segments representing various predictive variables. It offers several advantages, including practicality, ease of interpretation, and effective and accurate predictive capabilities, providing clinicians with an intuitive prediction tool that facilitates its use in clinical practice (Balachandran et al., 2015).

## 2 Materials and methods

### 2.1 Study design and participants

We conducted a retrospective analysis, gathering data from a total of 587 individuals who had suffered strokes and were subsequently admitted to the Rehabilitation Department of Dongguan Hospital, Guangzhou University of Traditional Chinese Medicine. This data collection period spanned from September 2021 to October 2024. The following inclusion criteria were established: (1) adhering to the diagnostic criteria for cerebrovascular disease outlined in the Fourth National Conference (Chinese Medical Association Neurology Branch, 2019) and diagnosed using CT and verified by MRI scans; (2) an age span of 18 to 90 years. The exclusion criteria comprised: (1) individuals with transient ischemic attacks; (2) individuals with a history of upper limb surgery; (3) patients with other severe comorbid conditions, including but not limited to malignant tumors, severe cardiovascular diseases, severe liver and biliary diseases, renal failure; (4) patients with significantly missing required case data; (5) patients who had experienced fever or infection in the past two weeks.

Finally, 376 stroke patients were enrolled. They were classified during their hospitalization into the training cohort (264 cases) and validation cohort (112 cases) at a ratio of 7:3. Discrimination was performed to determine whether the stroke patients had CRPS based on the diagnostic criteria published in 2024 (Abd-Elsayed et al., 2024). This study was conducted in accordance with the principles of the Declaration of Helsinki. The research protocol was approved by the Ethics Committee of Dongguan Hospital, Guangzhou University of Traditional Chinese Medicine (PJ [2025] No.2). Since this retrospective observational study did not infringe upon patient privacy and posed no risk to patient safety, informed consent was waived by the Ethics Committee. To protect patient confidentiality, the data from this study will not be made publicly available.

### 2.2 Clinical data acquisition

Predictive variables for patients with post-stroke CRPS were identified through an analysis of existing literature and clinical experience. Data were carefully gathered from the electronic medical records of the patients, which included: (1) Demographic information: Gender, age, income (low income, middle income, high income), smoking history, alcohol consumption history, medical history (hypertension, diabetes, coronary heart disease, hyperlipidemias, cervical spondylosis), previous stroke, previous shoulder diseases (chronic shoulder pain, shoulder trauma),type of stroke (hemorrhagic, infarction, or a combination of both), lesion location (cerebral cortex, cerebellum, thalamus, basal ganglia, brainstem), affected side of hemiplegia (left side, right side), muscle strength of the affected upper limb (0–3 levels, 4–5 levels), treatment (surgical treatment, conservative treatment), sleep disorders, and comorbid depression. (2) Laboratory indicators: White blood cells (WBC), hemoglobin (HBG), neutrophil percentage (NEUT%), C-reactive protein (CRP), prealbumin (PAB), albumin (ALB), uric acid (UA), fasting blood glucose (FBG), glycosylated hemoglobin (HbA1c), and D-dimer. (3) Barthel Index: This index was utilized to evaluate the patients’ activities of daily living (ADL). It comprises 10 aspects and categorizes scores into three groups: independent (100 points), essential self-care (61 to 99 points), and poor self-care ability (below 60 points). A higher score indicates greater independence and lower dependence. The primary outcome measured was whether or not the patient developed post-stroke CRPS. (4) The National Institutes of Health Stroke Scale (NIHSS) consists of 15 items, with a total score ranging from 0 to 42. Higher scores indicate more severe neurological impairment. The scores are classified as follows: a score of less than 5 indicates mild impairment; 5 to 15, moderate impairment; and 16 or greater, severe impairment.

### 2.3 Statistical analysis

Statistical analysis was primarily performed using R software (version 4.2.1) and SPSS software (IBM version 26.0). Descriptive statistics were conducted on 376 participants. Summarize categorical data using frequency (*n*) and percentage (%). Continuous variables with a normal distribution were expressed as mean ± standard deviation (SD), while continuous variables with a non-normal distribution were expressed as the median and interquartile range. Categorical variables are compared using chi-square analysis, while continuous variables are analyzed using the Mann-Whitney test.

The creation and application of the nomogram model involve three main steps. First, to mitigate multicollinearity and overfitting while simplifying the model and selecting variables, LASSO regression was employed, running 10-fold cross-validation to normalize the included variables and identify the optimal lambda value (Sauerbrei et al., 2007). Seven non-zero coefficients were used to determine the independent predictive features (Alhamzawi and Ali, 2018). Second, statistically significant variables were chosen to build the nomogram model through multivariable logistic regression analysis (Su et al., 2021). Third, the effectiveness of the nomogram was assessed through calibration plots and receiver operating characteristic (ROC), where the Area Under the ROC Curve (AUROC) value exceeding 0.7 demonstrates strong discrimination by the model. Decision curve analysis (DCA) and clinical impact curves (CIC) were employed to assess the clinical utility of the nomogram (Vickers et al., 2008).

## 3 Results

### 3.1 Study flow diagram

**FIGURE 1:**
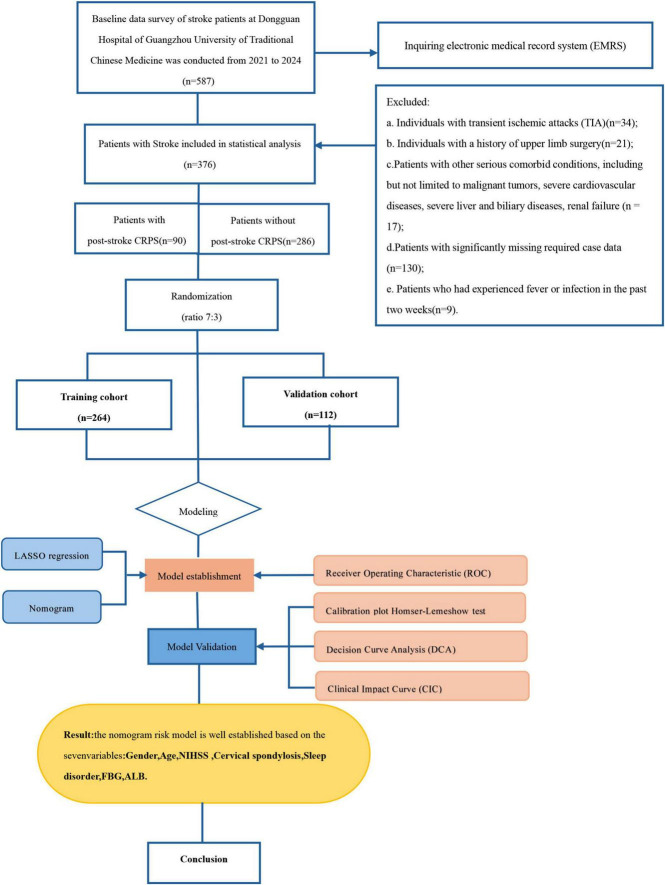
Flowchart.

The flowchart is illustrated in Figure 1. Out of the 587 stroke patients, 211 did not meet the inclusion criteria. Ultimately, a total of 376 individuals participated in the study.

### 3.2 Patient characteristics

The study included 376 stroke patients, of whom 286 (76.1%) were diagnosed with non-post-stroke CRPS, and 90 (23.9%) were diagnosed with post-stroke CRPS. Table 1 displays the differences between the two groups in terms of demographic data, clinical features, relevant scoring scales, and laboratory indicators.

**TABLE 1:** Descriptive and other characteristics of participants in training and validation group.

Variables	Training group				Validation group			
	**Total (*n* = 264)**	**Non-post CRPS (*n* = 207)**	**Post-stroke CRPS (*n* = 57)**	***P*-value**	**Total (*n* = 112)**	**Non-post CRPS (*n* = 79)**	**Post-stroke CRPS (*n* = 33)**	***P*-value**
**Demographics**								
Age, years, mean ± SD	62.47 ± 2.28	61.28 ± 12.46	66.82 ± 1.60	**0.002**	63 (52 ± 70)	60.48 ± 12.11	64.55 ± 10.37	0.095
**Gender, n (%)**								
Male	182 (68.9%)	154 (74.4%)	28 (49.1%)	**< 0.001**	79 (70.5%)	63 (79.7%)	16 (48.5%)	**0.001**
Female	82 (31.1%)	53 (25.6%)	29 (50.9%)		33 (29.5%)	16 (20.3%)	17 (51.5%)	
**Income, n (%)**								
Low income	63 (23.9%)	49 (23.7%)	14 (24.6%)	0.541	31 (27.7%)	23 (29.1%)	8 (24.2%)	0.209
Middle income	160 (60.6%)	129 (62.3%)	31 (54.4%)		64 (57.1%)	47 (59.5%)	17 (51.5%)	
High income	41 (15.5%)	29 (14%)	12 (21.1%)		17 (15.2%)	9 (11.4%)	8 (24.2%)	
**Medical history**								
Hypertension, *n* (%)	222 (84.1%)	175 (84.5%)	47 (82.5%)	0.703	88 (78.6%)	64 (81%)	24 (72.7%)	0.330
Diabetes, *n* (%)	99 (37.5%)	69 (33.3%)	30 (52.6%)	**0.008**	38 (33.9%)	28 (35.4%)	10 (30.3%)	0.600
CHD, *n* (%)	24 (9.1%)	18 (8.7%)	6 (10.5%)	0.670	14 (12.5%)	8 (10.1%)	6 (18.2%)	0.389
Hyperlipidemias, *n* (%)	61 (23.1%)	49 (23.7%)	12 (21.1%)	0.678	17 (15.2%)	12 (15.2%)	5 (15.2%)	1.000
Cervical spondylosis, *n* (%)	30 (11.4%)	12 (5.8%)	18 (31.6%)	**< 0.001**	11 (9.8%)	7 (8.9%)	4 (12.1%)	0.857
Previous stroke, *n* (%)	33 (12.5)	22 (10.6%)	11 (19.3%)	0.080	19 (17%)	12 (15.2%)	7 (21.2%)	0.439
**Previous shoulder disease, n (%)**								
Chronic shoulder pain	6 (2.3%)	4 (1.9%)	2 (3.5%)	**0.030**	2 (1.8%)	0 (0%)	2 (6.1%)	**< 0.001**
Shoulder trauma	8 (3%)	3 (1.4%)	5 (8.8%)		4 (3.6%)	0 (0%)	4 (12.1%)	
Smoking, *n* (%)	82 (31.1%)	64 (30.9%)	18 (31.6%)	0.924	32 (28.6%)	20 (25.3%)	12 (36.4%)	0.238
Alcohol drinking, *n* (%)	67 (25.4%)	53 (25.6%)	14 (24.6%)	0.873	23 (20.5%)	16 (20.3%)	7 (21.2%)	0.909
**Clinical features**								
**Types of stroke, n (%)**								
Infarction	200 (75.8%)	149 (72.0%)	51 (89.5%)	**0.022**	84 (75%)	55 (69.6%)	29 (87.9%)	0.084
Hemorrhage	58 (22.0%)	53 (25.6%)	5 (8.8%)		24 (21.4%)	21 (26.6%)	3 (9.1%)	
Infarction combined with hemorrhage	6 (2.3%)	5 (2.4%)	1 (1.8%)		4 (3.6%)	3 (3.8%)	1 (3%)	
**Location of occlusion, n (%)**								
Cerebral cortex	96 (36.4%)	74 (35.7%)	22 (38.6%)	0.907	39 (34.8%)	28 (35.4%)	11 (33.3%)	0.782
Cerebellum	11 (4.2%)	9 (4.3%)	2 (3.5%)		6 (5.4%)	3 (3.8%)	3 (9.1%)	
Thalamus	24 (9.1%)	18 (8.7%)	6 (10.5%)		9 (8%)	6 (7.6%)	3 (9.1%)	
Basal ganglia	101 (38.3%)	82 (39.6%)	19 (33.3%)		39 (34.8%)	29 (36.7%)	10 (30.3%)	
Brainstem	32 (12.9%)	24 (11.6%)	8 (14%)		13 (16.7%)	13 (16.5%)	13 (16.6%)	
**Hemiplegic limbs, n (%)**								
Left	143 (54.2%)	108 (52.2%)	35 (61.4%)	0.216	60 (53.6%)	37 (46.8%)	23 (69.7%)	**0.027**
Right	121 (45.8%)	99 (47.8%)	22 (38.6%)		52 (46.4%)	42 (53.2%)	10 (30.3%)	
**MMT upper, n (%)**								
0–3 level	128 (48.5%)	104 (50.2%)	24 (42.1%)	0.276	61 (54.5%)	48 (60.8%)	13 (39.4%)	**0.038**
4–5 level	136 (51.5%)	103 (49.8%)	33 (57.9%)		51 (45.5%)	31 (39.2%)	20 (60.6%)	
**Treatment, n (%)**								
Surgical treatment	66 (25%)	57 (27.5%)	9 (15.8%)	0.070	27 (24.1%)	20 (25.3%)	7 (21.2%)	0.643
Conservative treatment	198 (75%)	150 (72.5%)	48 (84.2%)		85 (75.9%)	59 (74.7%)	20 (25.3%)	
Sleep disorder, *n* (%)	46 (17.4%)	31 (15%)	15 (26.3%)	**0.046**	25 (22.3%)	12 (15.2%)	13 (39.4%)	**0.005**
Comorbid depression, *n* (%)	24 (9.1%)	20 (9.7%)	4 (7%)	0.539	8 (7.1%)	5 (6.3%)	3 (9.1%)	0.908
**NIHSS score, n (%)**								
< 5	148 (56.1%)	109 (52.7%)	39 (68.4%)	**0.024**	52 (46.4%)	33 (41.8%)	19 (57.6%)	0.128
5–15	108 (40.9%)	90 (43.5%)	18 (31.6%)		55 (49.1%)	42 (53.2%)	13 (39.4%)	
≥ 16	8 (3%)	8 (3.9%)	0 (0.00%)		5 (4.5%)	4 (5.1%)	1 (3%)	
**ADL, n (%)**								
100	27 (10.2%)	18 (8.7%)	9 (15.8%)	0.127	11 (9.8%)	7 (8.9%)	4 (12.1%)	0.742
99–61	157 (59.5%)	123 (59.4%)	34 (59.6%)		66 (58.9%)	47 (59.5%)	19 (57.6%)	
< 60	80 (30.3%)	66 (31.9%)	14 (24.6%)		35 (31.3%)	25 (31.6%)	10 (30.3%)	
**Inspection results**								
White blood cell, mean ± SD	6.96 ± 1.96	6.91 ± 1.90	7.17 ± 2.17	0.379	7.46 ± 2.25	6.98 ± 1.92	8.61 ± 2.59	**< 0.001**
Hemoglobin, mean ± SD	132.42 ± 17.04	133.05 ± 16.63	130.10 ± 18.42	0.247	131.30 ± 17.52	130.92 ± 15.92	132.22 ± 21.12	0.722
NEUT%, median (IQR)	64.25 (57.83–70.28)	63.7 (57.6–69.7)	66.2 (60.5–70.95)	0.054	65.5 (58.51–70.18)	64.2 (58.5–69.5)	68.4 (59.39–74.5)	0.075
C-reactive protein, median (IQR)	2.23 (1.00–5.40)	2.1 (0.99–5.05)	2.82 (1.01–6.79)	0.217	2.52 (1.26–7.49)	2.40 (1.22–6.40)	4.15 (1.50–9.16)	0.229
Prealbumin, mean ± SD	248.53 ± 47.58	250.57 ± 42.07	241.10 ± 63.62	0.184	240.11 ± 40.81	244.61 ± 37.67	229.30 ± 46.35	0.053
Albumin, mean ± SD	40.25 ± 4.14	40.01 ± 3.16	41.13 ± 6.53	0.070	39.65 ± 3.55	39.85 ± 3.10	39.19 ± 4.47	0.070
HbA1c, mean ± SD	6.21 ± 1.61	6.08 ± 1.47	6.67 ± 1.97	**0.013**	6.14 ± 1.27	6.01 ± 1.18	6.48 ± 1.40	0.373
Uric acid, mean ± SD	355.81 ± 97.95	360.04 ± 91.75	340.46 ± 117.39	0.182	366.12 ± 117.17	358.69 ± 117.25	383.92 ± 116.84	0.073
Fasting blood glucose, mean ± SD	5.90 ± 1.61	5.59 ± 1.19	7.02 ± 2.30	**< 0.001**	5.70 ± 1.32	5.48 ± 0.99	6.24 ± 1.81	0.301
D-dimer, median (IQR)	0.44 (0.22–0.91)	0.42 (0.22–0.89)	0.51 (0.28–1.05)	0.064	0.49 (0.27–0.95)	0.44 (0.22–0.76)	0.57 (0.41–1.00)	**0.005**

CHD, coronary heart disease; MMT upper, manual muscle testing (affected upper limb); NIHSS, National Institute of Health Stroke Scale; ADL, activities of daily living; NEUT%, the neutrophil percentage; HbA1c, glycated hemoglobin; IQR, interquartile range; SD, standard deviation. *P*-values less than 0.05 are highlighted in bold.

### 3.3 Independent risk factors in the training set

**FIGURE 2:**
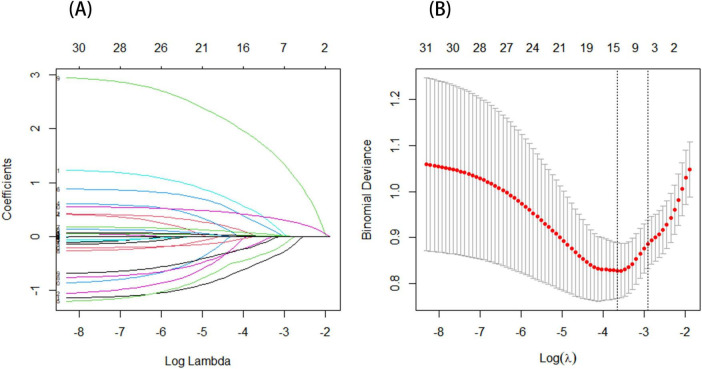
**(A)** The profile plot shows the LASSO coefficients for 31 features plotted against the log(lambda) sequence. Seven variables were chosen based on the optimal lambda. **(B)** A vertical dashed line is drawn to represent the value determined through 10-fold cross-validation. The optimal adjusted parameter log(λ) is displayed on the horizontal axis, while the deviance (binomial deviance) is shown on the vertical axis.

LASSO regression was carried out to determine the threatening elements linked to the development of CRPS among stroke patients included in the training set. This approach serves as a strong competitor for variable selection in Cox models (Sauerbrei et al., 2007). Determine the optimal regularization parameter λ based on 10-fold cross-validation and minimizing the standard error. The seven most relevant predictive factors were selected from a total of 31 correlated variables. These factors include gender, age, NIHSS score, cervical spondylosis, sleep disorders, FBG, and ALB (see Figures 2A, B for a visual representation of these findings).

### 3.4 Prediction model development

**FIGURE 3:**
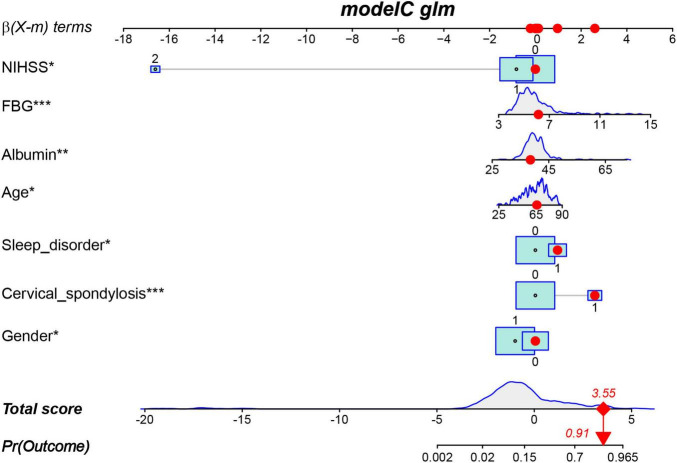
Nomogram for predicting CRPS in post-stroke patients.

The results of the multivariable logistic regression analysis indicate that gender, age, NIHSS score, cervical spondylosis, sleep disorder, FBG, and ALB are independent predictive factors for post-stroke CRPS (Table 2). All seven predictive variables demonstrated significant statistical differences. Therefore, based on these seven factors, a risk nomogram for post-stroke CRPS was developed (Figure 3).

**TABLE 2:** Multivariate logistic regression analysis.

Variables	OR	95% CI	*P*
**Gender**			
Male	0.413	0.189∼0.906	0.027
Age	1.044	1.006∼1.082	0.021
**NIHSS score**			
5–15	0.436	0.198∼0.964	0.040
Cervical spondylosis	13.447	4.764∼37.954	< 0.001
Sleep disorder	2.635	1.098∼6.324	0.030
Albumin	1.132	1.036∼1.237	0.006
FBG	1.739	1.368∼2.212	< 0.001

### 3.5 Validation of the nomogram

**FIGURE 4:**
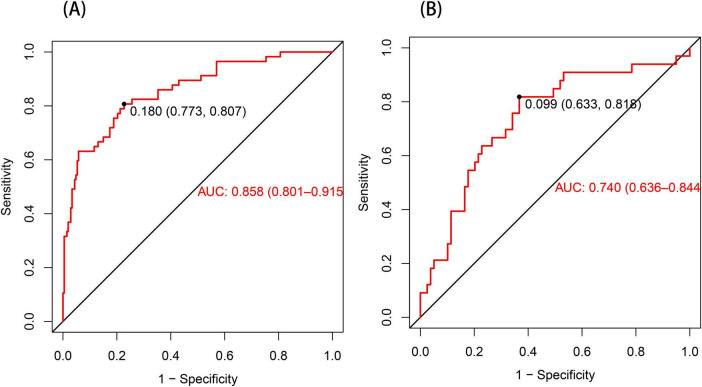
The ROC curve for the nomogram predicting post-stroke CRPS **(A)**. In the training set **(A)**, the AUC was 0.858 (95% CI: 0.801–0.915). When a cutoff point was set at a risk probability of 0.180, the specificity and sensitivity of the predicted results were 77.3% and 80.7%, respectively. In the validation set presented in **(B)**, the AUC was 0.740 (95% CI: 0.636–0.844), with a threshold of 0.099, a specificity of 0.633, and a sensitivity of 0.818.

**FIGURE 5:**
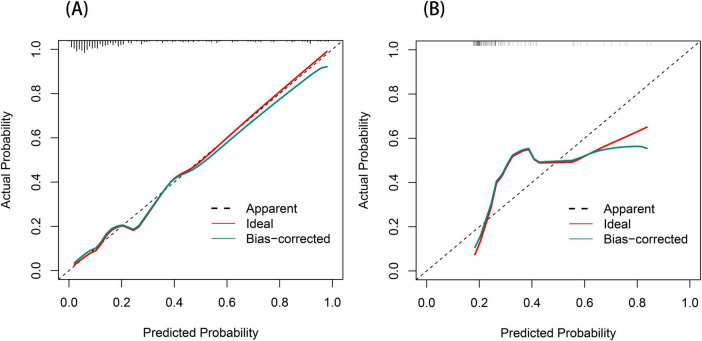
Calibration curve for the post-stroke CRPS nomogram **(A,B)**. The diagonal dashed line signifies perfect prediction by the optimal model. The proximity of the red solid line to this dashed line indicates the model’s predictive performance; the closer they are, the more accurate the predictions.

The ROC curve served as a tool for assessing the model’s predictive performance. The AUROC was 0.858 in the training set and 0.740 in the validation set (Figures 4A, B), indicating that the nomogram prediction model demonstrates good predictive capability. The Hosmer–Lemeshow goodness-of-fit test was employed to evaluate the calibration of the predictive model, and the calibration curve (Figures 5A, B) illustrated a strong correlation between the predicted probabilities and the actual occurrence of post-stroke CRPS in both the validation and training datasets.

### 3.6 Clinical efficacy of the nomogram

**FIGURE 6:**
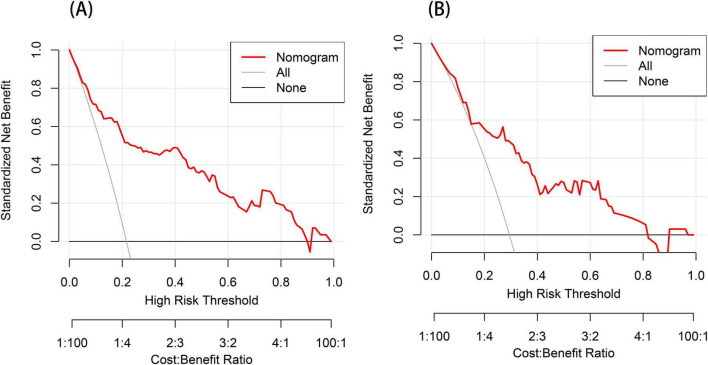
Decision curve analysis (DCA) for the post-stroke CRPS nomogram prediction **(A,B)**. The *y*-axis measures the net benefit obtained from using the model. The red line in the figure represents the clinical diagnostic model for post-stroke CRPS. In contrast, the black horizontal line (None line) and the gray diagonal line (All line) represent the extreme cases of “no intervention” and “all intervention,” respectively.

**FIGURE 7:**
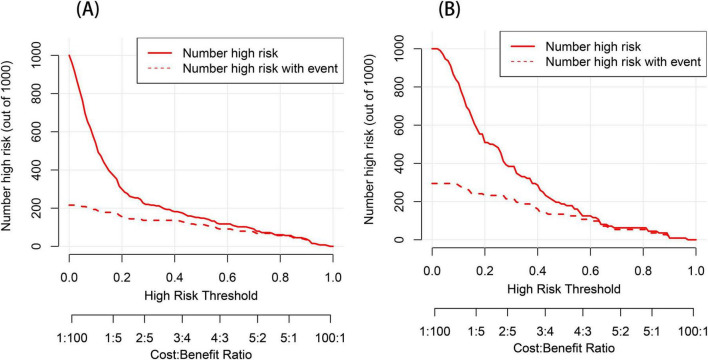
Clinical impact curve (CIC) analysis for the post-stroke CRPS nomogram prediction **(A,B)**. The *y*-axis represents the number of individuals at risk. The red line indicates the predicted number of events occurring for post-stroke CRPS, whereas the red dashed line depicts the actual occurrences.

We performed DCA to assess the clinical applicability of our nomogram. The DCA curves indicate that within a reasonable range of threshold probabilities, the nomogram model can provide clinical net benefit for patients in both the training group and the validation group (Figures 6A, B). Building on this, the CIC was plotted (Figures 7A, B) to assess the model’s clinical impact, showing that when the risk threshold exceeds 0.80, the number of positive events is closely aligned with the actual incidence of the condition.

## 4 Discussion

The etiology of post-stroke CRPS is complex, involving multiple factors, including neurological, circulatory, inflammatory, and psychological aspects. Manifestations include autonomic disturbances (such as skin temperature changes, color abnormalities, and sweating irregularities), sensory issues (pain and allodynia), and motor impairments (such as paralysis, tremors, and dystonia) (Borchers and Gershwin, 2017).

In most cases, only about one-fifth of patients are able to fully return to their previous level of regular activity (Matayoshi et al., 2009; Misidou and Papagoras, 2019), and this can even lead to irreversible permanent disability, serving as a barrier to rehabilitation and severely affecting patients’ quality of life. To date, there have been only a handful of research projects that have documented and reported on the various risk factors that are linked to the development of post-stroke CRPS (Katsura et al., 2024; Yu et al., 2023). To address this gap, we collected common clinical-related data variables and laboratory findings to establish a diagnostic prediction model, ultimately developing a simplified equation. The results indicate that a composite index of gender, age, NIHSS scores, cervical spondylosis, sleep disorders, FBG, and ALB demonstrates good predictive capability for the occurrence of post-stroke CRPS. As a result, this combined index may serve as a valuable asset that could significantly aid in the timely detection of post-stroke CRPS.

The age range for cases of post-stroke CRPS primarily falls between 45 and 75 years, with a common prevalence among individuals aged 56 to 65 (Hannan et al., 2013). Research conducted in the past has shown that age constitutes a significant and independent risk factor for the development of this condition (Zhang et al., 2004), with older individuals being more susceptible to developing CRPS. This could potentially be associated with the physiological alterations that take place in the skeletal structure, joints, and nervous system as individuals age. Additionally, regarding gender, it is noteworthy that our findings are inconsistent with those of van Velzen et al. (2019). The differences in sample size and the generally higher incidence of stroke in males compared to females (Carcel et al., 2020) may be the main reasons for this discrepancy. The underlying mechanisms are still unclear and require further investigation.

NIHSS score is a reliable indicator for assessing the severity of neurological impairment in stroke patients. Given the close association between neurological dysfunction and the development of post-stroke CRPS, existing studies have shown a positive correlation between NIHSS scores and the degree of neurological damage (Wang et al., 2025). Thus, stroke patients with higher NIHSS scores are more likely to develop CRPS.

Research has indicated that cervical spondylosis ought to be regarded as a potential risk factor for post-stroke CRPS (Steinbrocker, 1947). We propose that cervical spondylosis may influence the risk of developing CRPS through neural, vascular, and muscular pathways. Firstly, adverse neural stimulation can produce abnormal discharges, leading to persistent transmission of pain signals, which subsequently results in soft tissue spasms around the shoulder joint. Additionally, decreased peripheral nerve nourishment and blood circulation, along with abnormal vasodilation or vasoconstriction, can lead to localized hypoxia and an increase in pro-inflammatory substances (Parkitny et al., 2013), resulting in persistent pain, alterations in the temperature and color of skin, and swelling (Albrecht et al., 2006). Therefore, early diagnosis may be beneficial, and to ensure reliability, it will be essential to conduct further clinical research involving a more significant number of participants and data collected from multiple centers in the future.

Previous studies have found that stroke and sleep disturbances frequently coexist (Sonmez and Karasel, 2019), with one report indicating that 78% of stroke patients experience varying degrees of sleep disorders (Pasic et al., 2011). Additionally, some research has clearly shown that sleep problems are frequently observed in individuals suffering from shoulder injuries following a stroke (Al Battat et al., 2023; Küçükdeveci et al., 1996). A cohort study by Zhang et al. (2022) explored the effect of sleep disruptions on neurological functional outcomes after stroke, revealing a positive correlation between poor neurological function and sleep disturbances. Our study observed that sleep disturbances are an important predictive factor for the occurrence of CRPS following stroke. They can affect the autonomic nervous system, leading to sympathetic nerve dysfunction (Kato et al., 2000; Tobaldini et al., 2017), which in turn increases the risk of CRPS after stroke. Furthermore, sleep disturbances can trigger a series of inflammatory responses and oxidative stress, further damaging the nervous system (Atrooz and Salim, 2020) and significantly increasing the likelihood of CRPS in stroke patients.

Our study indicates that patients with post-stroke CRPS exhibit elevated fasting blood glucose (FBG) levels, with logistic regression modeling yielding an odds ratio (OR) of 1.739, indicating that FBG represents a significant risk factor for the development of the disease. One of the typical symptoms of post-stroke CRPS is pain. Studies have shown (Hok et al., 2024) that individual differences in spontaneous pain intensity in post-stroke CRPS are influenced by how insulin affects the resting-state functional connectivity (rsFC) of the prefrontal cortex. Insulin not only influences blood glucose levels but also alters neural network activity by regulating neurotransmitter release and affecting synaptic plasticity (Schwartz et al., 2024). Changes in FBG and insulin levels reflect the body’s response to metabolic load. Therefore, correcting glucose dysregulation may help achieve beneficial effects on the central nervous system, playing a crucial role in predicting CRPS in patients following a stroke. Continued research is needed to verify this potential mechanism.

We have innovatively discovered that albumin (ALB) can serve as a new independent predictive factor for post-stroke CRPS. Albumin levels are indicative of the body’s nutritional status; a decrease in albumin levels can catalyze muscle atrophy (Erdoğan et al., 2024; He et al., 2024; Tomata et al., 2022), leading to a loss of the “muscle pump” function (Zorowitz et al., 1996), which results in motor dysfunction and venous return obstruction. This can consequently cause swelling and pain in the hemiplegic upper limb, raising the possibility of post-stroke CRPS development.

Based on these results, we created a nomogram prediction model that provides a potential theoretical reference for assessing the risk of CRPS in patients following a stroke.

## 5 Conclusion

In this study, we identified significant correlations between gender, age, NIHSS scores, cervical spondylosis, sleep disorders, FBG, and ALB with the incidence of post-stroke CRPS. These predictive factors are readily available in clinical settings. Our simplified nomogram can assist clinicians in the early identification of stroke patients prone to developing CRPS, allowing for the initiation of empirical treatment to prevent or halt the progression of post-stroke CRPS. To validate the effectiveness of this new nomogram model, future large-scale prospective studies utilizing comprehensive data from multiple centers will be necessary.

## 6 Limitations

This study has certain limitations. Primarily, This is a single-center retrospective study, where both training and validation cohorts are derived from the same medical center, potentially limiting the model’s generalizability. Additionally, the study’s sample size is limited, necessitating future multicenter, prospective studies with larger cohorts to validate the model’s accuracy. Second, due to the historical data analysis nature of our study, data on sleep disturbance scores were lacking, preventing a more detailed analysis of the types and severity of sleep disturbances. Third, this study did not consider all possible risk factors and did not collect data on these factors, which may lead to bias in the results.

## Data Availability

The raw data supporting the conclusions of this article will be made available by the authors, without undue reservation.
